# Crystal structure of (*E*)-4-{1-[2-(car­bamo­thio­yl)hydrazin-1-yl­idene]ethyl}phenyl 4-methyl­benzoate

**DOI:** 10.1107/S2056989014026942

**Published:** 2015-01-01

**Authors:** Karthik Ananth Mani, Vijayan Viswanathan, S. Narasimhan, Devadasan Velmurugan

**Affiliations:** a Department of Chemistry, Asthagiri Herbal Research Foundation, Perungudi Industrial Estate, Perungudi, Chennai 600 096, India; b Centre of Advanced Study in Crystallography and Biophysics, University of Madras, Guindy Campus, Chennai 600 025, India

**Keywords:** crystal structure, thio­semicarbazones derivatives, biological activity, hydrogen bonding, ester

## Abstract

The asymmetric unit of the title compound, C_17_H_17_N_3_O_2_S, consists of two independent mol­ecules, *A* and *B*, with different conformations: in mol­ecule *A*, the dihedral angles between the central benzene ring and the pendant tolyl and carbamo­thio­ylhydrazono groups are 71.12 (9) and 5.95 (8)°, respectively. The corresponding angles in mol­ecule *B* are 50.56 (12) and 26.43 (11)°, respectively. Both mol­ecules feature an intra­molecular N—H⋯N hydrogen bond, which closes an *S*(5) ring. In the crystal, mol­ecules are linked by N—H⋯O, N—H⋯S and C—H⋯O hydrogen bonds, generating a three-dimensional network.

## Related literature   

For background to the biological activity of thio­semicarbazone derivatives, see: Reis *et al.* (2013 ▸); Fatondji *et al.* (2013 ▸); Sau *et al.* (2003 ▸); Seena *et al.* (2006 ▸).
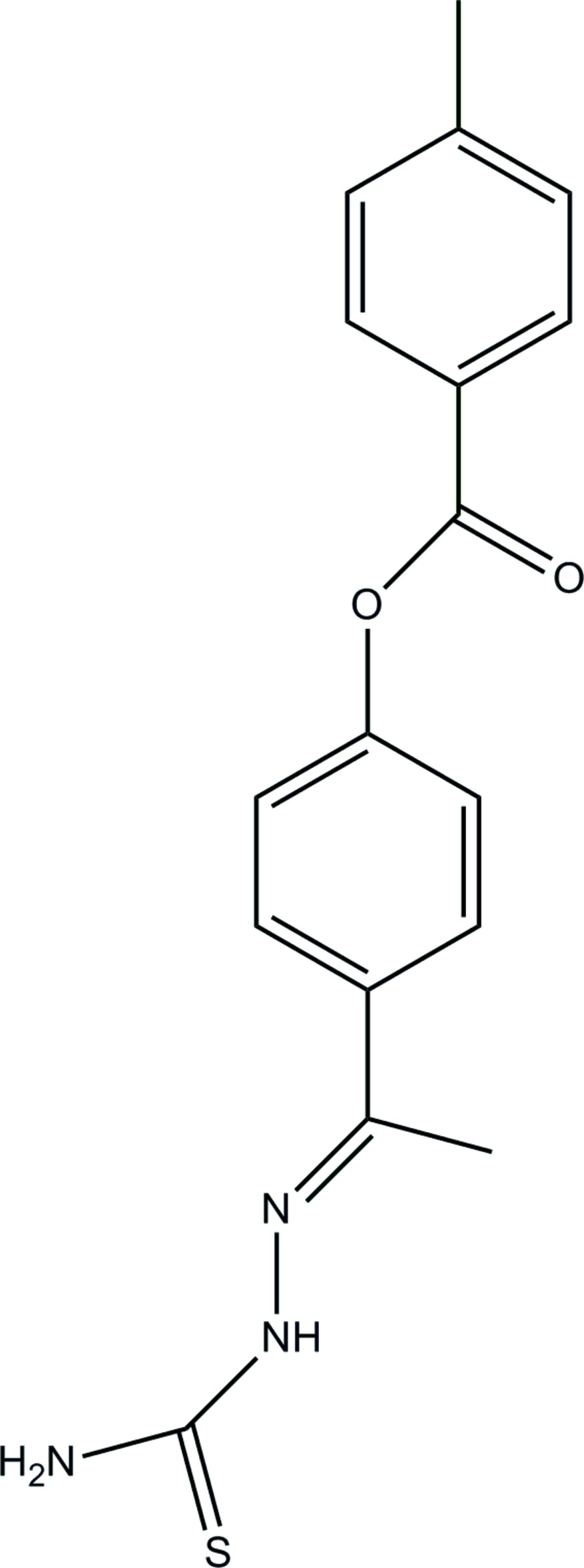


## Experimental   

### Crystal data  

C_17_H_17_N_3_O_2_S
*M* *_r_* = 327.40
Triclinic, 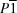

*a* = 8.068 (5) Å
*b* = 14.037 (5) Å
*c* = 15.221 (5) Å
α = 99.801 (5)°
β = 96.040 (5)°
γ = 98.533 (5)°
*V* = 1664.7 (13) Å^3^
*Z* = 4
Mo *K*α radiation
μ = 0.21 mm^−1^
*T* = 293 K
0.20 × 0.15 × 0.10 mm

### Data collection  

Bruker SMART APEXII CCD diffractometer
Absorption correction: multi-scan (*SADABS*; Bruker, 2008 ▸) *T*_min_ = 0.960, *T*_max_ = 0.980
25169 measured reflections
6809 independent reflections
5402 reflections with *I* > 2σ(*I*)
*R*_int_ = 0.030

### Refinement  

*R*[*F* ^2^ > 2σ(*F* ^2^)] = 0.040
*wR*(*F* ^2^) = 0.118
*S* = 0.99
6809 reflections
424 parameters
H atoms treated by a mixture of independent and constrained refinement
Δρ_max_ = 0.44 e Å^−3^
Δρ_min_ = −0.43 e Å^−3^

### 

Data collection: *APEX2* (Bruker, 2008 ▸); cell refinement: *SAINT* (Bruker, 2008 ▸); data reduction: *SAINT*; program(s) used to solve structure: *SHELXS97* (Sheldrick, 2008 ▸); program(s) used to refine structure: *SHELXL97* (Sheldrick, 2008 ▸); molecular graphics: *ORTEP-3 for Windows* (Farrugia, 2012 ▸); software used to prepare material for publication: *SHELXL97* and *PLATON* (Spek, 2009 ▸).

## Supplementary Material

Crystal structure: contains datablock(s) global, I. DOI: 10.1107/S2056989014026942/hb7334sup1.cif

Structure factors: contains datablock(s) I. DOI: 10.1107/S2056989014026942/hb7334Isup2.hkl

Supporting information file. DOI: 10.1107/S2056989014026942/hb7334Isup3.cml

**Fig. 1.**
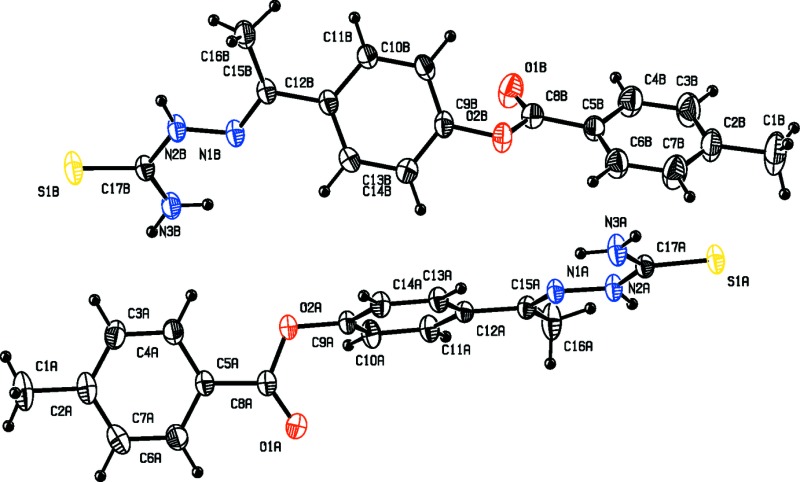
The molecular structure of the title compound, showing displacement ellipsoids drawn at 30% probability level.

**Fig. 2.**
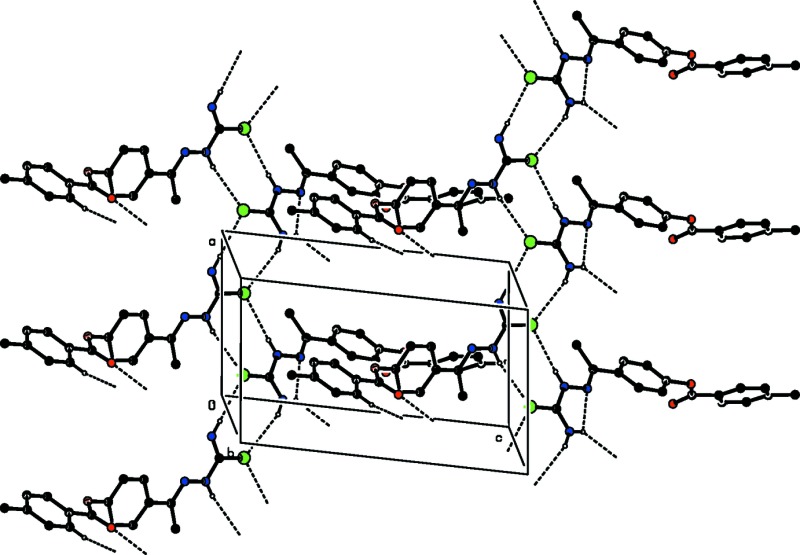
The crystal packing of the title compound viewed down b axis. H-atoms not involved in H-bonds have been excluded for clarity.

. DOI: 10.1107/S2056989014026942/hb7334fig1.tif
The mol­ecular structure of the title compound, showing displacement ellipsoids drawn at 30% probability level.

b . DOI: 10.1107/S2056989014026942/hb7334fig2.tif
The crystal packing of the title compound viewed down *b* axis. H-atoms not involved in H-bonds have been excluded for clarity.

CCDC reference: 1038319

Additional supporting information:  crystallographic information; 3D view; checkCIF report

## Figures and Tables

**Table 1:** Hydrogen-bond geometry (, )

*D*H*A*	*D*H	H*A*	*D* *A*	*D*H*A*
N3*A*H3*A*1N1*A*	0.86	2.28	2.634(3)	105
N3*B*H3*B*1N1*B*	0.86	2.25	2.600(3)	105
N2*A*H2*A*S1*B* ^i^	0.85(2)	2.66(2)	3.432(3)	153.5(8)
N2*B*H2*B*S1*A* ^ii^	0.86	2.67	3.513(3)	169
N3*A*H3*A*2S1*B* ^iii^	0.86	2.58	3.444(3)	178
N3*B*H3*B*1O1*A* ^iv^	0.86	2.42	3.159(3)	145
N3*B*N3B2S1*A* ^v^	0.86	2.78	3.456(3)	136
C6*A*H6*A*O1*B* ^vi^	0.93	2.53	3.354(3)	148

Symmetry codes: (i) 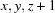; (ii) 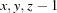; (iii) 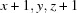; (iv) 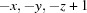; (v) 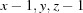; (vi) 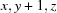.

## supplementary crystallographic information

### S1. Comment

Thiosemicarbazone and its derivatives are a class of O, N, S-tridentate donor ligands capable of stabilizing both higher and lower oxidation states of transition metal ions. The biological activities of these ligands are linked to their chelating ability with transition metal ions through phenol O, azomethine N and thiolate S atoms (Seena *et al.*, 2006). Thiosemicarbazones are significant intermediates in drugs synthesis, formation of metal complexes and heterocycles such as thiadiazolines preparation (Sau *et al.*, 2003). Thiosemicarbazones are reported as compounds which present significant antifungal activity. Their metal complexes also exhibit antifungal properties (Reis *et al.*, 2013).

Thiosemicarbnazones are inhibitors of DNA replication and also of many proteases. This inhibitory activity defends the level of interest given to them in the fight against microbial and parasitic diseases. Thiosemicarbazones have many biological activities such as antiviral, antibacterial, antitumor, African trypanosome (Fatondji *et al.*, 2013).

The title compound, C_17_H_17_O_2_N_3_S_1_, crystallizes in triclinic P -1 space group. The asymmetric unit of title compound contains two molecules which are shown in Fig.1. The acetophenone thiosemicarbazone fragment in molecule A is almost planar with maximum deviation -0.087 Å and in molecule B maximun deviation is -0.592 Å. The methylbenzoate (C1/C2-C8/O1/O2) and acetophenone thiosemicarbazone (C9-C16/N1/N2/C17/N3/S1) make a dihedral angle of 71.12 (1) ° in molecule A and 50.60 (1) ° in molecule B. The thiosemicarbazone group adopts an extended conformation,which can be seen from the torsion angle value of S1/C17/N2/N1 = -177.4 ° in molecule A and 174.1 ° in molecule B. The methylbenzoate and acetophenone thiosemicarbozone lie in a plane which is evidenced by the torsion angle value of C5/C8/O2/C9 = 179.4 ° in molecule A and 174.8 ° in molecule B.

The crystal structure features both intramolecular & intermolecular interactions of type N—H···N, C—H···O, C—H···N, N—H···S and N—H···O (Table. 1 & Fig. 2). In the crystal packing N—H···S type of intermolecular interaction shows R_2_2 (8) dimer formation.

### S2. Experimental

A 250-ml two neck RB flask was taken and fitted with condenser and an addition funnel. 0.5mol of 4- hydroxy acetophenone was taken and 200ml of chloroform was added to it with stirring. The reaction mixture was cooled at 5-10°c. 0.5mol of para-tolouyl chloride was added drop wise to the reaction mixture. Stirring was continued for another 15mins and 0.5mol of potassium carbonate was slowly added. Reaction was continued for 2 hours and monitored using TLC. The reaction mass was transferred into 1l beakers and washed twice with water (2x250ml). The chloroform layer was separated and washed with 10% NaOH solution (2x250ml). The chloroform layer was separated and dried with anhydrous sodium sulphate. The chloroform layer was filtered and concentrated under reduced pressure using rotary vacuum, cooled and hexane was added to it. Solid was precipitated, filtered and the product was air dried. Thiosemicarbazide (0.1mole) dissolved in 20 ml of 1N hydrochloric acid was added slowly in constant stirring to 4-Methyl-benzoic acid 4-acetyl-phenyl ester (0.1mole) dissolved in 50 ml of ethanol. After addition of thiosemicarbazide, novel 4-(1-(2-carbamothioylhydrazono)ethyl) phenyl 4-methylbenzoate (in solid form) was formed within 4 mins. The precipitate was filtered and washed with water, followed by Hexane wash and the product was air dried. After purification the compound was recrystallised from CHCl_3_ solution to yield colourless blocks.

### S3. Refinement

The hydrogen atoms were placed in calculated positions with C—H = 0.93Å to 0.96 Å & N—H = 0.85 Å to 0.86 Å and refined in the riding model with fixed isotropic displacement parameters:*U*_iso_(H) = 1.5*U*_eq_(C) for methyl group and *U*_iso_(H) = 1.2*U*_eq_(C) for other groups. The hydrogen atom H2Awas obtained from the difference fourier map.

### Crystal data

**Table:**

C_17_H_17_N_3_O_2_S	*Z* = 4
*M**_r_* = 327.40	*F*(000) = 688
Triclinic, *P*1	*D*_x_ = 1.306 Mg m^−^^3^
Hall symbol: -P 1	Mo *K*α radiation, λ = 0.71073 Å
*a* = 8.068 (5) Å	Cell parameters from 6809 reflections
*b* = 14.037 (5) Å	θ = 1.4–26.4°
*c* = 15.221 (5) Å	µ = 0.21 mm^−^^1^
α = 99.801 (5)°	*T* = 293 K
β = 96.040 (5)°	Block, colourless
γ = 98.533 (5)°	0.20 × 0.15 × 0.10 mm
*V* = 1664.7 (13) Å^3^	

### Data collection

**Table:**

Bruker SMART APEXII CCD diffractometer	6809 independent reflections
Radiation source: fine-focus sealed tube	5402 reflections with *I* > 2σ(*I*)
Graphite monochromator	*R*_int_ = 0.030
ω and φ scans	θ_max_ = 26.4°, θ_min_ = 1.4°
Absorption correction: multi-scan (*SADABS*; Bruker, 2008)	*h* = −10→10
*T*_min_ = 0.960, *T*_max_ = 0.980	*k* = −17→17
25169 measured reflections	*l* = −19→19

### Refinement

**Table:**

Refinement on *F*^2^	Secondary atom site location: difference Fourier map
Least-squares matrix: full	Hydrogen site location: inferred from neighbouring sites
*R*[*F*^2^ > 2σ(*F*^2^)] = 0.040	H atoms treated by a mixture of independent and constrained refinement
*wR*(*F*^2^) = 0.118	*w* = 1/[σ^2^(*F*_o_^2^) + (0.055*P*)^2^ + 0.6388*P*] where *P* = (*F*_o_^2^ + 2*F*_c_^2^)/3
*S* = 0.99	(Δ/σ)_max_ = 0.002
6809 reflections	Δρ_max_ = 0.44 e Å^−^^3^
424 parameters	Δρ_min_ = −0.43 e Å^−^^3^
0 restraints	Extinction correction: *SHELXL97* (Sheldrick, 2008), Fc^*^=kFc[1+0.001xFc^2^λ^3^/sin(2θ)]^-1/4^
Primary atom site location: structure-invariant direct methods	Extinction coefficient: 0.0180 (14)

### Special details

**Table:**

Geometry. All esds (except the esd in the dihedral angle between two l.s. planes) are estimated using the full covariance matrix. The cell esds are taken into account individually in the estimation of esds in distances, angles and torsion angles; correlations between esds in cell parameters are only used when they are defined by crystal symmetry. An approximate (isotropic) treatment of cell esds is used for estimating esds involving l.s. planes.
Refinement. Refinement of F^2^ against ALL reflections. The weighted R-factor wR and goodness of fit S are based on F^2^, conventional R-factors R are based on F, with F set to zero for negative F^2^. The threshold expression of F^2^ > 2sigma(F^2^) is used only for calculating R-factors(gt) etc. and is not relevant to the choice of reflections for refinement. R-factors based on F^2^ are statistically about twice as large as those based on F, and R- factors based on ALL data will be even larger.

### Fractional atomic coordinates and isotropic or equivalent isotropic displacement parameters (Å^2^)

**Table:**

	*x*	*y*	*z*	*U*_iso_*/*U*_eq_	
C1A	0.2654 (3)	0.3477 (2)	0.22085 (17)	0.0842 (8)	
H1A1	0.1730	0.3830	0.2150	0.126*	
H1A2	0.2578	0.2980	0.1680	0.126*	
H1A3	0.3704	0.3922	0.2276	0.126*	
C1B	0.2414 (5)	−0.5299 (3)	1.03242 (19)	0.1202 (12)	
H1B1	0.2019	−0.5983	1.0305	0.180*	
H1B2	0.1741	−0.4914	1.0675	0.180*	
H1B3	0.3575	−0.5132	1.0593	0.180*	
C2A	0.2576 (2)	0.30002 (16)	0.30253 (13)	0.0568 (5)	
C2B	0.2266 (3)	−0.50911 (19)	0.93750 (15)	0.0756 (7)	
C3A	0.3217 (3)	0.21517 (16)	0.30697 (13)	0.0602 (5)	
H3A	0.3704	0.1865	0.2586	0.072*	
C3B	0.1513 (4)	−0.58023 (18)	0.86641 (17)	0.0840 (8)	
H3B	0.1081	−0.6420	0.8766	0.101*	
C4A	0.3152 (2)	0.17175 (15)	0.38156 (12)	0.0524 (4)	
H4A	0.3582	0.1140	0.3828	0.063*	
C4B	0.1374 (3)	−0.56294 (16)	0.77975 (15)	0.0722 (6)	
H4B	0.0837	−0.6124	0.7324	0.087*	
C5A	0.2449 (2)	0.21369 (13)	0.45461 (11)	0.0432 (4)	
C5B	0.2031 (2)	−0.47226 (14)	0.76322 (12)	0.0510 (4)	
C6A	0.1808 (3)	0.29922 (14)	0.45138 (13)	0.0538 (5)	
H6A	0.1333	0.3283	0.5000	0.065*	
C6B	0.2807 (3)	−0.40036 (16)	0.83396 (14)	0.0653 (6)	
H6B	0.3265	−0.3391	0.8237	0.078*	
C7A	0.1873 (3)	0.34158 (15)	0.37588 (15)	0.0619 (5)	
H7A	0.1437	0.3991	0.3744	0.074*	
C7B	0.2913 (4)	−0.41833 (19)	0.92051 (15)	0.0806 (7)	
H7B	0.3429	−0.3685	0.9682	0.097*	
C8A	0.2332 (2)	0.17051 (13)	0.53614 (12)	0.0463 (4)	
C8B	0.1894 (3)	−0.45931 (14)	0.66842 (13)	0.0526 (4)	
C9A	0.2862 (2)	0.03688 (12)	0.60304 (11)	0.0458 (4)	
C9B	0.2889 (2)	−0.35490 (14)	0.57185 (12)	0.0491 (4)	
C10A	0.1370 (3)	−0.01089 (14)	0.62106 (12)	0.0533 (5)	
H10A	0.0367	−0.0128	0.5840	0.064*	
C10B	0.3512 (3)	−0.41679 (17)	0.50947 (14)	0.0652 (6)	
H10B	0.3822	−0.4744	0.5230	0.078*	
C11A	0.1362 (2)	−0.05646 (13)	0.69479 (12)	0.0482 (4)	
H11A	0.0345	−0.0888	0.7071	0.058*	
C11B	0.3679 (3)	−0.39335 (16)	0.42609 (14)	0.0607 (5)	
H11B	0.4132	−0.4348	0.3842	0.073*	
C12A	0.2847 (2)	−0.05474 (11)	0.75085 (10)	0.0382 (3)	
C12B	0.3183 (2)	−0.30912 (13)	0.40365 (11)	0.0436 (4)	
C13A	0.4345 (2)	−0.00642 (13)	0.72968 (11)	0.0457 (4)	
H13A	0.5356	−0.0044	0.7660	0.055*	
C13B	0.2575 (3)	−0.24762 (14)	0.46904 (13)	0.0542 (5)	
H13B	0.2255	−0.1900	0.4561	0.065*	
C14A	0.4361 (2)	0.03851 (13)	0.65589 (12)	0.0492 (4)	
H14A	0.5374	0.0696	0.6420	0.059*	
C14B	0.2433 (3)	−0.26986 (15)	0.55296 (13)	0.0571 (5)	
H14B	0.2031	−0.2274	0.5963	0.069*	
C15A	0.2819 (2)	−0.10194 (12)	0.83068 (10)	0.0396 (4)	
C15B	0.3277 (2)	−0.28611 (13)	0.31284 (11)	0.0445 (4)	
C16A	0.1160 (2)	−0.15187 (19)	0.84955 (14)	0.0677 (6)	
H16A	0.1354	−0.1916	0.8938	0.102*	
H16B	0.0556	−0.1926	0.7951	0.102*	
H16C	0.0506	−0.1034	0.8718	0.102*	
C16B	0.4547 (3)	−0.32317 (18)	0.25743 (14)	0.0677 (6)	
H16D	0.4031	−0.3458	0.1962	0.102*	
H16E	0.4948	−0.3763	0.2805	0.102*	
H16F	0.5479	−0.2712	0.2599	0.102*	
C17A	0.5770 (2)	−0.14183 (12)	1.00110 (10)	0.0393 (4)	
C17B	0.0977 (2)	−0.16131 (13)	0.17673 (11)	0.0435 (4)	
N1A	0.42539 (17)	−0.09698 (10)	0.87810 (9)	0.0409 (3)	
N1B	0.22145 (18)	−0.23287 (11)	0.28867 (10)	0.0463 (3)	
N2A	0.42746 (19)	−0.13852 (11)	0.95384 (9)	0.0447 (3)	
N2B	0.22622 (18)	−0.20308 (12)	0.20702 (10)	0.0484 (4)	
H2B	0.3087	−0.2110	0.1765	0.058*	
N3A	0.71545 (19)	−0.10356 (13)	0.97208 (11)	0.0579 (4)	
H3A1	0.7082	−0.0778	0.9247	0.070*	
H3A2	0.8130	−0.1043	1.0005	0.070*	
N3B	−0.03094 (19)	−0.15950 (12)	0.22271 (10)	0.0539 (4)	
H3B1	−0.0310	−0.1845	0.2705	0.065*	
H3B2	−0.1152	−0.1333	0.2051	0.065*	
O1A	0.1798 (2)	0.20548 (11)	0.60231 (9)	0.0701 (4)	
O1B	0.1080 (2)	−0.51644 (13)	0.60607 (10)	0.0786 (5)	
O2A	0.29057 (18)	0.08334 (9)	0.52831 (8)	0.0545 (3)	
O2B	0.27954 (19)	−0.37271 (10)	0.65930 (8)	0.0604 (4)	
S1A	0.58069 (6)	−0.19659 (4)	1.09103 (3)	0.05114 (15)	
S1B	0.10592 (6)	−0.11364 (5)	0.08234 (4)	0.06621 (18)	
H2A	0.340 (3)	−0.1537 (15)	0.9779 (13)	0.054 (6)*	

### Atomic displacement parameters (Å^2^)

**Table:**

	*U*^11^	*U*^22^	*U*^33^	*U*^12^	*U*^13^	*U*^23^
C1A	0.0860 (17)	0.1031 (19)	0.0727 (15)	0.0020 (14)	0.0047 (13)	0.0580 (14)
C1B	0.164 (3)	0.142 (3)	0.0603 (16)	0.014 (3)	0.0099 (18)	0.0494 (18)
C2A	0.0477 (10)	0.0717 (12)	0.0541 (11)	−0.0003 (9)	−0.0006 (9)	0.0336 (10)
C2B	0.0962 (18)	0.0885 (16)	0.0474 (12)	0.0142 (14)	0.0099 (11)	0.0288 (11)
C3A	0.0628 (12)	0.0775 (13)	0.0464 (10)	0.0130 (10)	0.0130 (9)	0.0244 (10)
C3B	0.121 (2)	0.0648 (14)	0.0683 (15)	−0.0018 (14)	0.0186 (15)	0.0302 (12)
C4A	0.0574 (11)	0.0596 (11)	0.0474 (10)	0.0178 (9)	0.0098 (8)	0.0219 (8)
C4B	0.0984 (18)	0.0589 (12)	0.0547 (12)	−0.0026 (12)	0.0111 (12)	0.0112 (10)
C5A	0.0419 (9)	0.0502 (9)	0.0393 (9)	0.0065 (7)	0.0013 (7)	0.0171 (7)
C5B	0.0548 (11)	0.0576 (11)	0.0451 (10)	0.0137 (9)	0.0096 (8)	0.0173 (8)
C6A	0.0566 (11)	0.0566 (11)	0.0528 (11)	0.0133 (9)	0.0079 (9)	0.0196 (9)
C6B	0.0821 (15)	0.0571 (11)	0.0556 (12)	0.0014 (11)	0.0076 (11)	0.0180 (9)
C7A	0.0600 (12)	0.0589 (11)	0.0740 (14)	0.0127 (10)	0.0019 (10)	0.0343 (10)
C7B	0.105 (2)	0.0794 (16)	0.0478 (12)	−0.0046 (14)	−0.0020 (12)	0.0091 (11)
C8A	0.0498 (10)	0.0516 (10)	0.0393 (9)	0.0082 (8)	0.0027 (8)	0.0156 (7)
C8B	0.0569 (11)	0.0562 (11)	0.0465 (10)	0.0126 (9)	0.0063 (9)	0.0125 (9)
C9A	0.0638 (11)	0.0450 (9)	0.0325 (8)	0.0151 (8)	0.0046 (8)	0.0141 (7)
C9B	0.0497 (10)	0.0621 (11)	0.0392 (9)	0.0107 (8)	0.0034 (8)	0.0204 (8)
C10A	0.0545 (11)	0.0632 (11)	0.0424 (10)	0.0086 (9)	−0.0082 (8)	0.0204 (8)
C10B	0.0807 (15)	0.0749 (13)	0.0616 (12)	0.0418 (12)	0.0230 (11)	0.0395 (11)
C11A	0.0460 (10)	0.0565 (10)	0.0426 (9)	0.0053 (8)	−0.0018 (8)	0.0182 (8)
C11B	0.0759 (14)	0.0702 (12)	0.0534 (11)	0.0376 (11)	0.0235 (10)	0.0298 (10)
C12A	0.0453 (9)	0.0385 (8)	0.0313 (8)	0.0099 (7)	0.0017 (7)	0.0078 (6)
C12B	0.0397 (9)	0.0512 (9)	0.0430 (9)	0.0083 (7)	0.0022 (7)	0.0190 (7)
C13A	0.0441 (9)	0.0533 (10)	0.0411 (9)	0.0086 (8)	0.0000 (7)	0.0164 (7)
C13B	0.0663 (12)	0.0508 (10)	0.0517 (11)	0.0187 (9)	0.0061 (9)	0.0205 (8)
C14A	0.0528 (11)	0.0536 (10)	0.0446 (10)	0.0075 (8)	0.0083 (8)	0.0189 (8)
C14B	0.0742 (13)	0.0561 (11)	0.0438 (10)	0.0170 (10)	0.0097 (9)	0.0110 (8)
C15A	0.0407 (9)	0.0462 (9)	0.0325 (8)	0.0075 (7)	0.0022 (7)	0.0106 (7)
C15B	0.0409 (9)	0.0515 (9)	0.0442 (9)	0.0069 (7)	0.0030 (7)	0.0204 (8)
C16A	0.0453 (11)	0.1051 (17)	0.0563 (12)	−0.0030 (11)	−0.0034 (9)	0.0447 (12)
C16B	0.0735 (14)	0.0900 (15)	0.0585 (12)	0.0372 (12)	0.0215 (11)	0.0388 (11)
C17A	0.0379 (8)	0.0475 (9)	0.0350 (8)	0.0091 (7)	0.0028 (7)	0.0138 (7)
C17B	0.0372 (9)	0.0533 (9)	0.0430 (9)	0.0068 (7)	0.0027 (7)	0.0200 (7)
N1A	0.0416 (8)	0.0509 (8)	0.0341 (7)	0.0096 (6)	0.0029 (6)	0.0186 (6)
N1B	0.0410 (8)	0.0596 (9)	0.0423 (8)	0.0074 (7)	0.0019 (6)	0.0241 (7)
N2A	0.0367 (8)	0.0647 (9)	0.0380 (7)	0.0081 (7)	0.0036 (6)	0.0257 (7)
N2B	0.0385 (8)	0.0706 (10)	0.0449 (8)	0.0136 (7)	0.0073 (6)	0.0307 (7)
N3A	0.0373 (8)	0.0918 (12)	0.0540 (9)	0.0095 (8)	0.0052 (7)	0.0417 (9)
N3B	0.0493 (9)	0.0763 (11)	0.0493 (9)	0.0240 (8)	0.0136 (7)	0.0326 (8)
O1A	0.1059 (12)	0.0702 (9)	0.0497 (8)	0.0356 (9)	0.0302 (8)	0.0248 (7)
O1B	0.0948 (12)	0.0818 (10)	0.0504 (9)	−0.0068 (9)	0.0023 (8)	0.0117 (8)
O2A	0.0786 (9)	0.0569 (7)	0.0376 (6)	0.0248 (7)	0.0118 (6)	0.0216 (6)
O2B	0.0743 (9)	0.0671 (8)	0.0420 (7)	0.0061 (7)	0.0067 (6)	0.0227 (6)
S1A	0.0424 (2)	0.0724 (3)	0.0461 (3)	0.0105 (2)	0.00270 (19)	0.0335 (2)
S1B	0.0452 (3)	0.1100 (5)	0.0638 (3)	0.0247 (3)	0.0145 (2)	0.0588 (3)

### Geometric parameters (Å, º)

**Table:**

C1A—C2A	1.511 (3)	C10A—H10A	0.9300
C1A—H1A1	0.9600	C10B—C11B	1.379 (3)
C1A—H1A2	0.9600	C10B—H10B	0.9300
C1A—H1A3	0.9600	C11A—C12A	1.390 (2)
C1B—C2B	1.518 (3)	C11A—H11A	0.9300
C1B—H1B1	0.9600	C11B—C12B	1.387 (3)
C1B—H1B2	0.9600	C11B—H11B	0.9300
C1B—H1B3	0.9600	C12A—C13A	1.392 (2)
C2A—C3A	1.376 (3)	C12A—C15A	1.480 (2)
C2A—C7A	1.385 (3)	C12B—C13B	1.383 (3)
C2B—C3B	1.360 (4)	C12B—C15B	1.479 (2)
C2B—C7B	1.381 (3)	C13A—C14A	1.379 (2)
C3A—C4A	1.379 (2)	C13A—H13A	0.9300
C3A—H3A	0.9300	C13B—C14B	1.378 (3)
C3B—C4B	1.377 (3)	C13B—H13B	0.9300
C3B—H3B	0.9300	C14A—H14A	0.9300
C4A—C5A	1.383 (3)	C14B—H14B	0.9300
C4A—H4A	0.9300	C15A—N1A	1.284 (2)
C4B—C5B	1.379 (3)	C15A—C16A	1.496 (3)
C4B—H4B	0.9300	C15B—N1B	1.283 (2)
C5A—C6A	1.382 (3)	C15B—C16B	1.490 (3)
C5A—C8A	1.476 (2)	C16A—H16A	0.9600
C5B—C6B	1.368 (3)	C16A—H16B	0.9600
C5B—C8B	1.480 (3)	C16A—H16C	0.9600
C6A—C7A	1.383 (3)	C16B—H16D	0.9600
C6A—H6A	0.9300	C16B—H16E	0.9600
C6B—C7B	1.379 (3)	C16B—H16F	0.9600
C6B—H6B	0.9300	C17A—N3A	1.318 (2)
C7A—H7A	0.9300	C17A—N2A	1.350 (2)
C7B—H7B	0.9300	C17A—S1A	1.6798 (16)
C8A—O1A	1.195 (2)	C17B—N3B	1.312 (2)
C8A—O2A	1.362 (2)	C17B—N2B	1.343 (2)
C8B—O1B	1.195 (2)	C17B—S1B	1.6883 (17)
C8B—O2B	1.357 (2)	N1A—N2A	1.3774 (18)
C9A—C10A	1.366 (3)	N1B—N2B	1.3795 (19)
C9A—C14A	1.375 (3)	N2A—H2A	0.85 (2)
C9A—O2A	1.4053 (19)	N2B—H2B	0.8600
C9B—C10B	1.363 (3)	N3A—H3A1	0.8600
C9B—C14B	1.367 (3)	N3A—H3A2	0.8600
C9B—O2B	1.403 (2)	N3B—H3B1	0.8600
C10A—C11A	1.383 (2)	N3B—H3B2	0.8600
			
C2A—C1A—H1A1	109.5	C11B—C10B—H10B	120.3
C2A—C1A—H1A2	109.5	C10A—C11A—C12A	121.17 (17)
H1A1—C1A—H1A2	109.5	C10A—C11A—H11A	119.4
C2A—C1A—H1A3	109.5	C12A—C11A—H11A	119.4
H1A1—C1A—H1A3	109.5	C10B—C11B—C12B	121.18 (19)
H1A2—C1A—H1A3	109.5	C10B—C11B—H11B	119.4
C2B—C1B—H1B1	109.5	C12B—C11B—H11B	119.4
C2B—C1B—H1B2	109.5	C11A—C12A—C13A	117.70 (15)
H1B1—C1B—H1B2	109.5	C11A—C12A—C15A	120.75 (15)
C2B—C1B—H1B3	109.5	C13A—C12A—C15A	121.55 (14)
H1B1—C1B—H1B3	109.5	C13B—C12B—C11B	117.59 (16)
H1B2—C1B—H1B3	109.5	C13B—C12B—C15B	120.94 (15)
C3A—C2A—C7A	118.00 (17)	C11B—C12B—C15B	121.47 (17)
C3A—C2A—C1A	121.3 (2)	C14A—C13A—C12A	121.33 (16)
C7A—C2A—C1A	120.7 (2)	C14A—C13A—H13A	119.3
C3B—C2B—C7B	118.1 (2)	C12A—C13A—H13A	119.3
C3B—C2B—C1B	120.7 (2)	C14B—C13B—C12B	121.41 (17)
C7B—C2B—C1B	121.2 (2)	C14B—C13B—H13B	119.3
C2A—C3A—C4A	121.4 (2)	C12B—C13B—H13B	119.3
C2A—C3A—H3A	119.3	C9A—C14A—C13A	119.26 (17)
C4A—C3A—H3A	119.3	C9A—C14A—H14A	120.4
C2B—C3B—C4B	121.6 (2)	C13A—C14A—H14A	120.4
C2B—C3B—H3B	119.2	C9B—C14B—C13B	119.39 (18)
C4B—C3B—H3B	119.2	C9B—C14B—H14B	120.3
C3A—C4A—C5A	120.29 (18)	C13B—C14B—H14B	120.3
C3A—C4A—H4A	119.9	N1A—C15A—C12A	116.24 (14)
C5A—C4A—H4A	119.9	N1A—C15A—C16A	125.21 (15)
C3B—C4B—C5B	120.0 (2)	C12A—C15A—C16A	118.55 (14)
C3B—C4B—H4B	120.0	N1B—C15B—C12B	114.65 (16)
C5B—C4B—H4B	120.0	N1B—C15B—C16B	125.04 (16)
C6A—C5A—C4A	118.99 (16)	C12B—C15B—C16B	120.30 (15)
C6A—C5A—C8A	118.13 (16)	C15A—C16A—H16A	109.5
C4A—C5A—C8A	122.88 (16)	C15A—C16A—H16B	109.5
C6B—C5B—C4B	119.00 (18)	H16A—C16A—H16B	109.5
C6B—C5B—C8B	123.52 (18)	C15A—C16A—H16C	109.5
C4B—C5B—C8B	117.45 (18)	H16A—C16A—H16C	109.5
C5A—C6A—C7A	120.05 (19)	H16B—C16A—H16C	109.5
C5A—C6A—H6A	120.0	C15B—C16B—H16D	109.5
C7A—C6A—H6A	120.0	C15B—C16B—H16E	109.5
C5B—C6B—C7B	120.3 (2)	H16D—C16B—H16E	109.5
C5B—C6B—H6B	119.9	C15B—C16B—H16F	109.5
C7B—C6B—H6B	119.9	H16D—C16B—H16F	109.5
C6A—C7A—C2A	121.27 (19)	H16E—C16B—H16F	109.5
C6A—C7A—H7A	119.4	N3A—C17A—N2A	117.40 (15)
C2A—C7A—H7A	119.4	N3A—C17A—S1A	122.89 (13)
C6B—C7B—C2B	121.0 (2)	N2A—C17A—S1A	119.68 (13)
C6B—C7B—H7B	119.5	N3B—C17B—N2B	117.76 (15)
C2B—C7B—H7B	119.5	N3B—C17B—S1B	122.49 (13)
O1A—C8A—O2A	122.20 (16)	N2B—C17B—S1B	119.75 (13)
O1A—C8A—C5A	125.62 (17)	C15A—N1A—N2A	117.89 (14)
O2A—C8A—C5A	112.18 (15)	C15B—N1B—N2B	119.05 (15)
O1B—C8B—O2B	122.65 (18)	C17A—N2A—N1A	119.45 (14)
O1B—C8B—C5B	125.32 (18)	C17A—N2A—H2A	116.6 (14)
O2B—C8B—C5B	112.02 (16)	N1A—N2A—H2A	123.1 (14)
C10A—C9A—C14A	121.03 (16)	C17B—N2B—N1B	117.74 (14)
C10A—C9A—O2A	120.61 (16)	C17B—N2B—H2B	121.1
C14A—C9A—O2A	118.34 (16)	N1B—N2B—H2B	121.1
C10B—C9B—C14B	120.89 (17)	C17A—N3A—H3A1	120.0
C10B—C9B—O2B	121.41 (17)	C17A—N3A—H3A2	120.0
C14B—C9B—O2B	117.57 (17)	H3A1—N3A—H3A2	120.0
C9A—C10A—C11A	119.48 (17)	C17B—N3B—H3B1	120.0
C9A—C10A—H10A	120.3	C17B—N3B—H3B2	120.0
C11A—C10A—H10A	120.3	H3B1—N3B—H3B2	120.0
C9B—C10B—C11B	119.50 (18)	C8A—O2A—C9A	116.43 (13)
C9B—C10B—H10B	120.3	C8B—O2B—C9B	117.77 (15)
			
C7A—C2A—C3A—C4A	0.7 (3)	C10B—C11B—C12B—C15B	−176.86 (19)
C1A—C2A—C3A—C4A	179.79 (19)	C11A—C12A—C13A—C14A	0.2 (3)
C7B—C2B—C3B—C4B	−0.7 (5)	C15A—C12A—C13A—C14A	−179.16 (16)
C1B—C2B—C3B—C4B	−179.7 (3)	C11B—C12B—C13B—C14B	−1.3 (3)
C2A—C3A—C4A—C5A	−0.7 (3)	C15B—C12B—C13B—C14B	178.00 (18)
C2B—C3B—C4B—C5B	1.0 (4)	C10A—C9A—C14A—C13A	−1.9 (3)
C3A—C4A—C5A—C6A	0.3 (3)	O2A—C9A—C14A—C13A	179.83 (15)
C3A—C4A—C5A—C8A	179.62 (17)	C12A—C13A—C14A—C9A	1.1 (3)
C3B—C4B—C5B—C6B	−0.4 (4)	C10B—C9B—C14B—C13B	1.3 (3)
C3B—C4B—C5B—C8B	177.9 (2)	O2B—C9B—C14B—C13B	177.27 (17)
C4A—C5A—C6A—C7A	0.1 (3)	C12B—C13B—C14B—C9B	−0.6 (3)
C8A—C5A—C6A—C7A	−179.26 (17)	C11A—C12A—C15A—N1A	179.65 (16)
C4B—C5B—C6B—C7B	−0.6 (4)	C13A—C12A—C15A—N1A	−1.0 (2)
C8B—C5B—C6B—C7B	−178.8 (2)	C11A—C12A—C15A—C16A	0.2 (3)
C5A—C6A—C7A—C2A	−0.1 (3)	C13A—C12A—C15A—C16A	179.55 (18)
C3A—C2A—C7A—C6A	−0.3 (3)	C13B—C12B—C15B—N1B	−26.1 (2)
C1A—C2A—C7A—C6A	−179.40 (19)	C11B—C12B—C15B—N1B	153.15 (19)
C5B—C6B—C7B—C2B	1.0 (4)	C13B—C12B—C15B—C16B	153.3 (2)
C3B—C2B—C7B—C6B	−0.3 (4)	C11B—C12B—C15B—C16B	−27.4 (3)
C1B—C2B—C7B—C6B	178.7 (3)	C12A—C15A—N1A—N2A	178.84 (14)
C6A—C5A—C8A—O1A	−3.7 (3)	C16A—C15A—N1A—N2A	−1.8 (3)
C4A—C5A—C8A—O1A	177.0 (2)	C12B—C15B—N1B—N2B	176.61 (14)
C6A—C5A—C8A—O2A	175.97 (16)	C16B—C15B—N1B—N2B	−2.8 (3)
C4A—C5A—C8A—O2A	−3.4 (2)	N3A—C17A—N2A—N1A	0.6 (2)
C6B—C5B—C8B—O1B	−171.8 (2)	S1A—C17A—N2A—N1A	−177.45 (12)
C4B—C5B—C8B—O1B	9.9 (3)	C15A—N1A—N2A—C17A	174.57 (16)
C6B—C5B—C8B—O2B	7.3 (3)	N3B—C17B—N2B—N1B	−5.9 (2)
C4B—C5B—C8B—O2B	−170.9 (2)	S1B—C17B—N2B—N1B	174.13 (12)
C14A—C9A—C10A—C11A	1.5 (3)	C15B—N1B—N2B—C17B	169.44 (17)
O2A—C9A—C10A—C11A	179.68 (16)	O1A—C8A—O2A—C9A	−0.9 (3)
C14B—C9B—C10B—C11B	−0.2 (3)	C5A—C8A—O2A—C9A	179.38 (14)
O2B—C9B—C10B—C11B	−176.00 (19)	C10A—C9A—O2A—C8A	75.9 (2)
C9A—C10A—C11A—C12A	−0.2 (3)	C14A—C9A—O2A—C8A	−105.8 (2)
C9B—C10B—C11B—C12B	−1.7 (3)	O1B—C8B—O2B—C9B	−6.1 (3)
C10A—C11A—C12A—C13A	−0.6 (3)	C5B—C8B—O2B—C9B	174.76 (16)
C10A—C11A—C12A—C15A	178.70 (17)	C10B—C9B—O2B—C8B	−57.6 (3)
C10B—C11B—C12B—C13B	2.4 (3)	C14B—C9B—O2B—C8B	126.5 (2)

### Hydrogen-bond geometry (Å, º)

**Table:**

*D*—H···*A*	*D*—H	H···*A*	*D*···*A*	*D*—H···*A*
N3*A*—H3*A*1···N1*A*	0.86	2.28	2.634 (3)	105
N3*B*—H3*B*1···N1*B*	0.86	2.25	2.600 (3)	105
N2*A*—H2*A*···S1*B*^i^	0.85 (2)	2.66 (2)	3.432 (3)	153.5 (8)
N2*B*—H2*B*···S1*A*^ii^	0.86	2.67	3.513 (3)	169
N3*A*—H3*A*2···S1*B*^iii^	0.86	2.58	3.444 (3)	178
N3*B*—H3*B*1···O1*A*^iv^	0.86	2.42	3.159 (3)	145
N3*B*—N3B2···S1*A*^v^	0.86	2.78	3.456 (3)	136
C6*A*—H6*A*···O1*B*^vi^	0.93	2.53	3.354 (3)	148

Symmetry codes: (i) *x*, *y*, *z*+1; (ii) *x*, *y*, *z*−1; (iii) *x*+1, *y*, *z*+1; (iv) −*x*, −*y*, −*z*+1; (v) *x*−1, *y*, *z*−1; (vi) *x*, *y*+1, *z*.
