# Supplementary figures and images for: Crystal structure of (E)-4-{1-[2-(car­bamo­thio­yl)hydrazin-1-yl­idene]ethyl}phenyl 4-methyl­benzoate
Source: Acta Crystallogr E Crystallogr Commun. 2015 Jan 1;71(Pt 1):o43–4. doi: 10.1107/S2056989014026942 (PMC4331904; doi:10.1107/S2056989014026942)

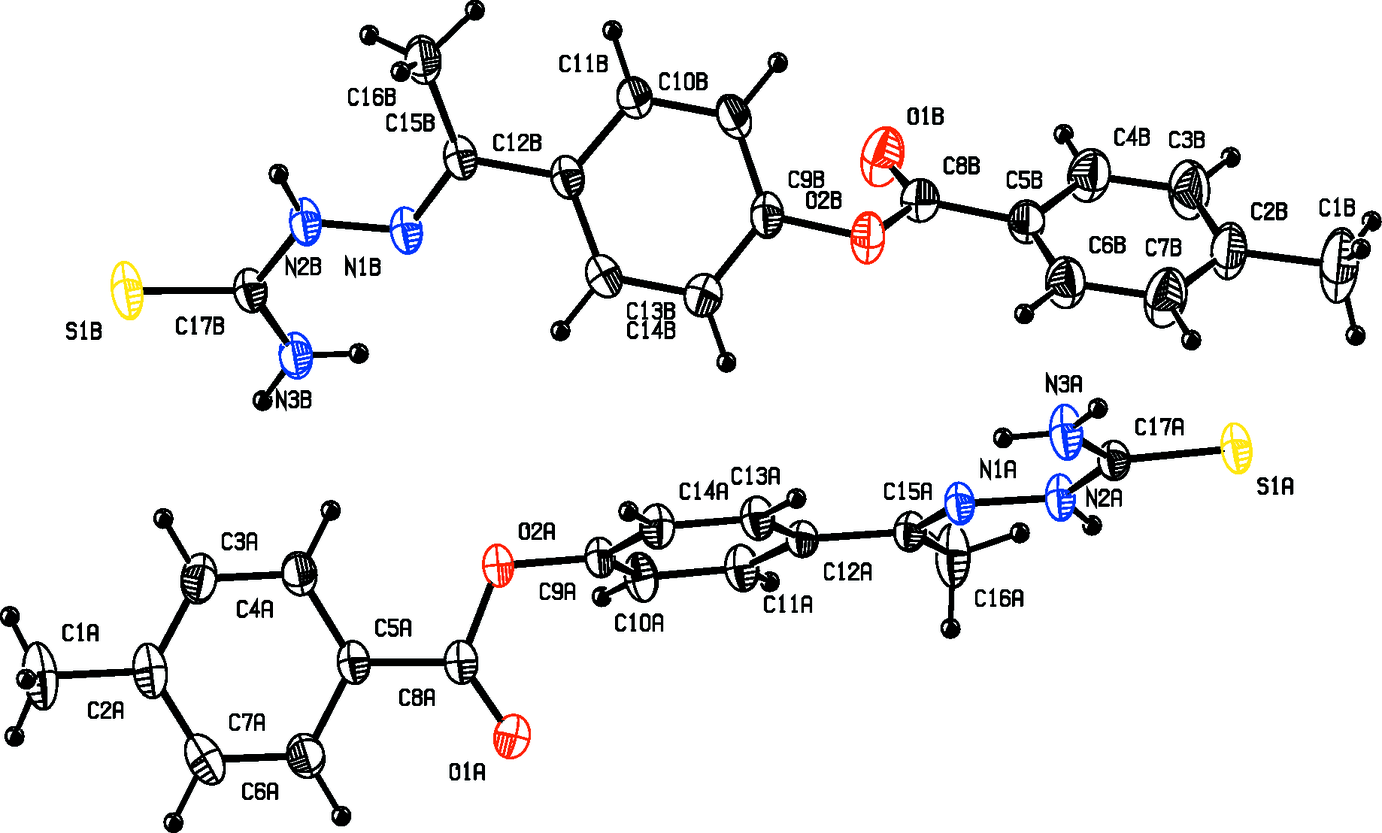

Supplement: Supplementary file 4 [file e-71-00o43-fig1.tif]

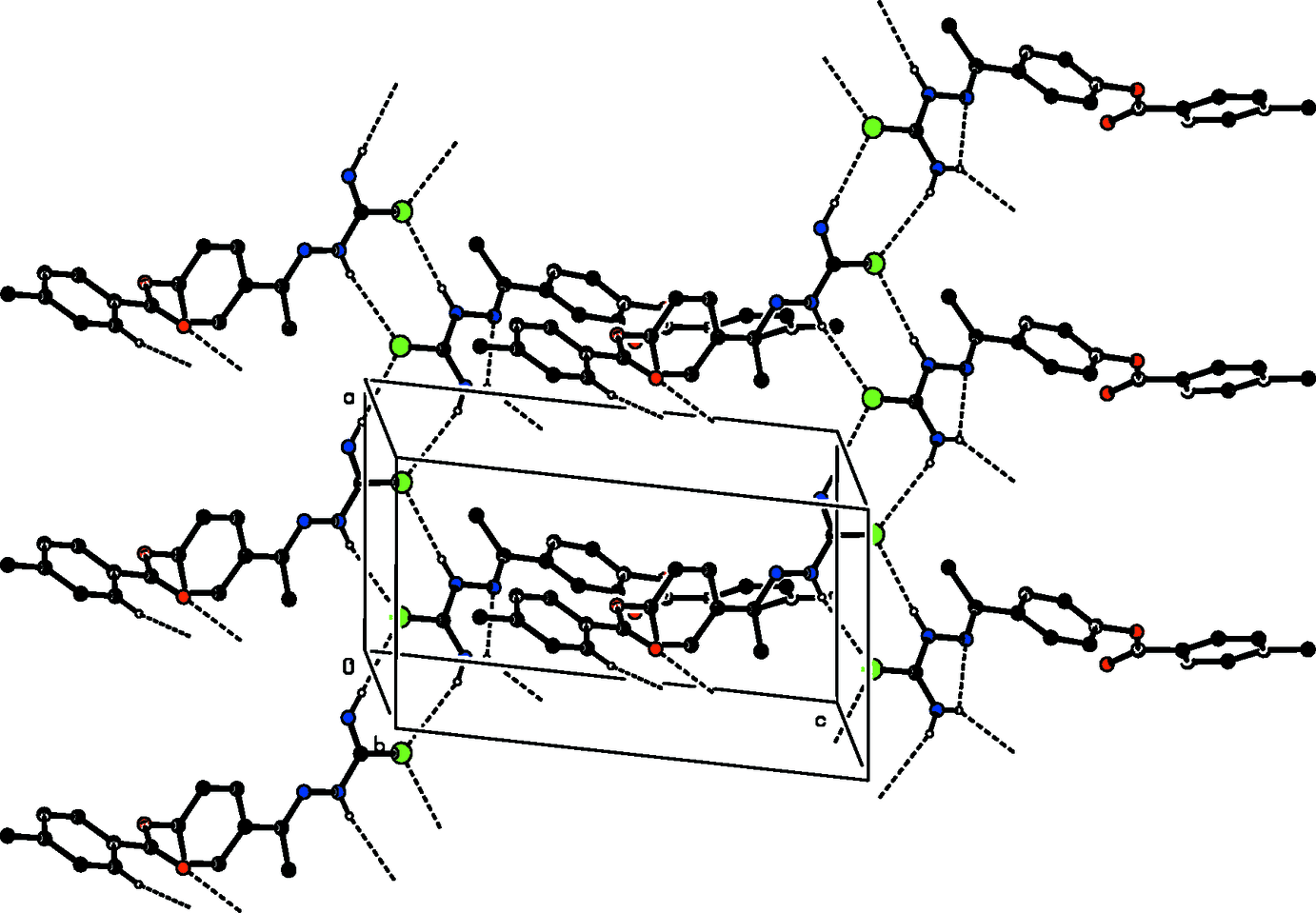

Supplement: Supplementary file 5 [file e-71-00o43-fig2.tif]
